# Comparative study of an externship program versus a corporate-academic cooperation program for enhancing nursing competence of graduating students

**DOI:** 10.1186/1472-6920-13-108

**Published:** 2013-08-14

**Authors:** Chien-Ning Tseng, Chia-Ju Hsieh, Kee-Hsin Chen, Meei-Fang Lou

**Affiliations:** 1 Department of Nursing, College of Medicine, National Taiwan University, 1, Jen-Ai Rd., Sec. 1, Taipei 10051, Taiwan, R.O.C; 2 Cardinal Tien College of Healthcare & Management, 112, Minzu Rd. Xindian Dist., New Taipei City 231, Taiwan, R.O.C; 3 Taipie Medical University-Municipal Wan Fang Hospital, 111, Sec. 3, Xinglong Rd. Wenshan Dist., Taipei City 116, Taiwan, R.O.C

**Keywords:** Clinical competence, Corporate-academic cooperation program, Nursing education, Nursing students, Retention

## Abstract

**Background:**

New graduates report intense stress during the transition from school to their first work settings. Managing this transition is important to reduce turnover rates. This study compared the effects of an externship program and a corporate-academic cooperation program on enhancing junior college students’ nursing competence and retention rates in the first 3 months and 1 year of initial employment*.*

**Methods:**

This two-phase study adopted a pretest and posttest quasi-experimental design. All participants were graduating students drawn from a 5-year junior nursing college in Taiwan. There were 19 and 24 students who participated in the phase I externship program and phase II corporate-academic cooperation program, respectively. The nursing competence of the students had to be evaluated by mentors within 48 hours of practicum training and after practicum training. The retention rate was also surveyed at 3 months and 1 year after beginning employment.

**Results:**

Students who participated in the corporate-academic cooperation program achieved a statistically significant improvement in nursing competence and retention rates relative to those who participated in the externship program (*p <* 0.01 and *p <* 0.05, respectively).

**Conclusions:**

The corporate-academic cooperation program facilitates the transition of junior college nursing students into independent staff nurses, enhances their nursing competence, and boosts retention rates.

## Background

Nursing is a stressful occupation, especially for newly graduated students [1]. The transition period from student to new nurse has been described as “the most stressful time” for many newly graduated nurses [2]. Several qualitative studies have examined this issue. Stress is primarily related to sudden changes in role; gaps between school and the clinical workplace; lack of professional knowledge and skills [3,4]; lack of confidence in providing independent care; lack of familiarity with medical devices or procedures for machines or tests; responding to ambiguous or unfamiliar orders and diagnoses [3,5]; poor interactions with patients, their families and other professionals [5,6]; and insufficient support from other nurses [3,7]. All of the stressors mentioned above assume that graduating students have limited nursing competence. A high-stress working environment without the required competence can generate a high turnover rate for newly graduated nurses. According to a nationwide survey of the National Union of Nurses Associations in the Republic of China in 2005, the first-year turnover rate for newly graduated nurses during initial employment reached 57.7%, which was 2–3 times higher than the average turnover rate for registered nurses (RNs) [8].

The preceptorship program was implemented many years ago to address the adaptation and retention of newly graduated nurses in hospitals in Taiwan. However, the nurse to patient ratio in Taiwan can range from 8-11-to-1 during daytime shifts [9], which can limit the effectiveness of preceptorships to improve the retention of newly graduated nurses. Thus, graduating nursing students’ transition to RN requires an additional preparation period before official entry into the clinical setting to allow adaptation during the transitional period. As a result, the externship program (EP) has been proposed to facilitate a smooth progression from graduating nursing students to RNs in nursing schools in Taiwan. However, the effectiveness of the EP in improving nursing competence and the retention rate are unknown.

Compared with institutes of technology or university-based nursing schools, junior nursing colleges face greater challenges related to graduates with insufficient professional competency as well as high turnover rates after employment. Factors contributing to this phenomenon include the following: (1) Changes in Taiwan’s educational policy. Beginning in 2000, the government abolished all vocational nursing schools and approved the transition of junior colleges to institutes of technology. To fulfill the qualifications necessary for this advancement, nursing schools are required to recruit personnel trained at graduate schools in Taiwan and abroad as teachers in technical and vocational institutions. These teachers possess educational credentials of a high level, but they generally lack practical experience and skills. Thus, the relevant nursing schools ultimately train students who fail to satisfy the employment demands of care organizations and hospitals [10,11]. (2) Insufficient clinical practicum venues or opportunities at the regional hospital level (and higher levels). Because of the requirements of Taiwan’s Joint Commission on Hospital Accreditation regarding the educational level of nursing personnel that is required in teaching and regional hospitals, nursing students from non-hospital-affiliated junior nursing colleges experience greater difficulty in obtaining clinical practicum opportunities at teaching and regional hospitals relative to university nursing students. These obstacles increase the likelihood that students of junior nursing colleges will lack sufficient opportunities to deal with emergencies and patients with severe diseases [12]. (3) Significant discrepancies between clinical practicum and clinical work field. Nursing students carry a reduced number of cases relative to the actual clinical care ratio, and students are not allocated night shifts. Therefore, nursing students tend to lack a comprehensive familiarity with the arrangement of work procedures for nurses and with patient conditions. Consequently, nursing students are unable to achieve a global perspective regarding the nursing profession [13,14]. The inadequate professional competency of nursing students when entering the workforce is exacerbated by gaps in school education, clinical practicum, institutions and regulations.

New graduates should be alerted to the potential for professional adjustment issues before course completion so they will be better equipped and more confident to take on such a professional position [15]. Current literature also addresses the need for a more collaborative effort in the integration of theory and clinical practice, as well as the need for education and services to minimize the difficulties that new graduates encounter during the transition from school to clinic [7,16]. In 2003, the Ministry of Education and The Council of Labor Affairs of the Republic of China vigorously promoted the adoption of a “corporate-academic cooperation program, CACP” among colleges. This program includes courses, seminars and practical training, and it was planned jointly by academia and industry [17]. Such programs have been implemented across many professions, including engineering and business fields. The outcomes of such programs include increased teaching resources at schools, reduction of the gap between schools and industry and integration of the learning experiences of students. The promotion of CACP by government organizations provides an opportunity for junior nursing colleges to bridge the gaps in school education, clinical practicum, institutions and regulations. Therefore, we provided a pilot CACP that replaced the usual EP in our school, with the aim of enhancing nursing competence and retention rates among the participating students.

The purpose of this study was to answer the following research question:

1. Is there better effect on nursing competence of graduating students before practicum in CACP group than those in EP group?

2. Is there better effect on nursing competence of graduating students after practicum in CACP group than those in EP group?

3. Is there better retention rate during the first 3 months of initial employment of nursing students in CACP group than those in EP group?

4. Is there better retention rate during the first 1 year of initial employment of nursing students in CACP group than those in EP group?

## Methods

### Design

Due to limited funding for research and the requirement of sponsors for the number of trained students, a quasi-experimental, non-equal sample size design conducted in two phases with pre/post-test evaluations was executed as follows: in phase 1, the EP comparison group was conducted from January 2006 to December 2007; and in phase II, the CACP intervention group was conducted from January 2007 to December 2008. For both the EP and CACP groups, the additional practicum was conducted in the same hospitals, including 1 medical center and 2 regional teaching hospitals in Taipei, Taiwan. No junior college nursing student participants reported clinical experience, and no participants paid extra tuition for the 2 programs.

### Comparative group (EP group)

The comparative group (EP group) originally consisted of senior nursing students who wanted to work as nurses immediately after graduation. All graduating students who participated in the EP group received practical training after they had completed all courses (195 credits) and practica (25 credits) typically required for students in the school. Among these courses, we chose 4 restricted elective courses in the final year of college, including ‘Gerontological Nursing’ , ‘Critical Care Nursing’ , ‘Introduction to Nursing Diagnosis’ and ‘Clinical Case Studies’. These courses were taught by the nursing faculty only, given the lack of coordination between the school and hospitals in course design and instruction, and they were required to be completed by the EP students prior to the practicum. Finally, students completed an additional 2-credit practicum course (Practicum in Comprehensive Clinical Nursing) after the end of course but before graduation.

This practicum course was conducted in the final school year for students who wished to obtain real-world work experience in a training setting, based on selections by the students regarding where they wanted to be employed following graduation. Each training unit only placed 1 student. Students served unlicensed assistive roles in a wide variety of specialties and settings, ranging from primary care to critical care. The training program lasted 4 weeks, 20 days and 160 hours, including 3 days (24 hours) for new personnel training provided by the hospitals. Each student received a one-on-one, planned training program devised by a mentor who was a senior competent nurse in the training setting. Students functioned as full-time registered nurses under the direct supervision of these mentors. The goals of the training were to allow graduating students to perform nursing duties in a real-world working situation and to become familiar with the hospital environment, job content, professional knowledge and required skills and attitudes. Students in this practicum were also expected to take full responsibility for the nursing care of one-half of an RN’s patient load in the unit by the end of 4 consecutive weeks of work.

Given that the term “preceptor” merely implies a teaching relationship [18], we selected the designation of “mentor” rather than “preceptor” to suggest a more long-term developmental relationship (as students transitioned to new staff). This distinction also differentiated the mentorship from the preceptorship of new training staff in the hospital setting. For nurses to be mentors of graduating students in this study, they had to fulfill the following criteria: (1) a minimum of 3 years of experience in the current unit; (2) favorable performance evaluations approved by the employed institution; (3) accomplishment of professional competencies and annual training requirements on the clinical ladder systems; (4) experience as a trained preceptor in new staff training; and (5) willingness to provide learning experiences for students. Based on the above criteria, we are confident that the mentors recruited in this study were qualified to exercise the professional judgment needed to assess a graduating student’s competence, regardless of whether the student was appropriate to stay in the training unit. Individual training units only placed 1 graduating student each; thus, the inter-rater reliability was not assessed (see discussion section). To ensure that the mentors were familiar with Schwirian's 6-dimension scale as an evaluation method for determining the nursing competence of a graduating student, we held a forum to present detailed instructions for the scale and provided a printed manual for each mentor.

### Intervention group

The intervention group completed the same courses and practicum typically required for students prior to the additional clinical practicum. Additionally, the intervention had the following characteristics:

#### 1. Established collaborative partnerships between the school and hospitals

At multiple points before beginning the intervention, the researchers (also nursing faculty) visited the hospitals and each clinical setting where the graduating students could be placed. The visits were designed to promote collaborative relationships between the school and hospitals and to provide an opportunity for the researchers to gather more information regarding the philosophy of the nursing administrator and the quality of the nursing personnel, as well as the culture, layout, policies and hiring and training procedures for new nursing personnel in those hospitals. Furthermore, the researchers also explained the goals, content, schedules and procedures for the practicum (or courses); the expected role of the mentor; and a list of clinical skills that students had acquired from previous classroom teaching and practical training, with the aim of helping the nursing administrator and mentors to better understand the program.

#### 2. Courses jointly designed and taught by nursing faculty and nursing managers

We invited qualified clinical instructors to participate in the design and instruction of the curriculum and practicum. In total, 7 courses worth 13 credits, including 3 newly added courses (‘Medical Terminology’ , ‘Career Education’ and a seminar) and the 4 former, restricted elective courses described as above, and all 7 courses were jointly designed and taught by the nursing faculty and nursing managers, not as comparative group taught by the nursing faculty only, given the lack of coordination between the school and hospitals in course design and instruction. Besides, the seminar was designed to help each student to obtain valuable experience from the senior registered nurses, to write a resume and to prepare for job interviews.

#### 3. Practicum arrangement

Prior to the additional practicum, all participants in the intervention group were required to complete all courses and the practicum typically required for students in the school, in addition to the 7 restricted elective courses. Participants in the intervention group received the same practicum design as those in the comparative group, i.e., 2 credits/4 weeks/20 days/160 hours practicum and one-on-one direct supervision by the mentor.

### Participants

The participants were graduating students drawn from a 5-year associate’s degree nursing program of a college in New Taipei city. The sample size was calculated based on the results of a literature review. The function used to estimate the sample size was adopted from Barcikowski and Robey [19]. The μ_1_, i.e., the average nursing competence score in Schwirian’s research, was 135.83, whereas μ_2_, the average expected nursing competence in our sample, was estimated as 158.52. The value of σ^2^, which represents the squared variance for the average expected nursing competence score for this population, was 484.94 [20]. The 2-tailed alpha level was set to 0.05, and the desired power was set to 0.8. Finally, a minimum of 15 subjects a group is desirable for research purposes (N=(2×σ^2^)÷(μ_2_-μ_1_)× f(α, β)=14.88). In the EP and CACP groups, 19 and 24 students met the criteria, respectively, and participated in either the EP or CACP under the supervision of their respective mentors within the training settings.

### Ethical considerations

The study was approved by the institutional review board of Cardinal Tien College of Healthcare & Management. Details of the program including the courses, seminars and practical training were thoroughly explained to the graduating students, legal guardians, school and hospital administrators and mentors. Written consent was obtained from every enrolled graduating student, their legal guardians and their mentors. Information regarding personal names, practice units and service units was encoded to protect the privacy of all participants.

### Data collection

All participating students were evaluated for nursing competence at T0 (48 hours within practicum training) and T1 (after practicum training) by their mentors. The retention rates were surveyed at 3 months and 1 year after employment was initiated.

### Outcome measures

Data analysis and statistics were conducted using the following 2 outcome measures:

#### 1. ***Nursing competence***

The 6-dimension scale of nursing competence developed by Schwirian [20] is one of the few scales that has been used for assessing educational preparation and has been extensively tested for validity and reliability [21,22]. The Chinese revised version, translated by Chen et al., consists of 46 questions divided into the following 6 subscales: clinical care (CC), teaching/collaboration (T/C), planning/evaluation (P/E), interpersonal relations/communication (TPR/C), professional development (PD) and leadership. The instrument uses a 5-point scale, with responses ranging from complete inability to perform the tasks (1) to each task was performed very well (5), and the overall scale score is calculated by summing all items. A higher score indicates superior clinical care capacity. The previously reported content validity index was 0.86, and Cronbach’s α for internal consistency was 0.96 [23].

#### 2. Retention rate

The retention rate was defined as the proportion of graduates who remained in their first hospital of employment and were employed as a nurse for at least 1 year.

### Data analysis

Data were analyzed using SPSS, version 16 (SPSS, Inc., Chicago, IL, USA), and were reviewed and double entered to ensure accuracy. Descriptive and univariate statistics, including chi-squared (for categorical variables) and independent 2-sample t-tests (for continuous variables) were used to describe the sample and to compare the outcomes between the 2 groups. To investigate whether students in the CACP group demonstrated higher scores in nursing competence relative to the EP group at T1, a multivariate analysis of covariance (MANCOVA) was used with the level of the 6 nursing competence variables at baseline applied as a covariate. Furthermore, we calculated the power of the study required to detect observed nursing competence in the 2 trial groups.

## Results

### Baseline characteristics of hospitals and mentors

In the EP and CACP groups, 19 and 24 students were enrolled and 19 and 23 students completed the programs, respectively. At baseline, the hospital characteristics and demographics of the mentors in the 2 groups were examined by chi-squared analysis and independent t-tests to ensure homogeneity. Table 1 shows that there were no significant differences in the baseline characteristics of the 2 groups. However, 39.5% and 60.5% of the graduating students were placed at the medical center and regional teaching hospitals for the practicum, respectively. Most students were trained in medical wards (51.2%). Forty-three RNs participated in this program as mentors and were characterized as follows: mean age of 28.86 years (SD= 2.51), most were college-educated (33, 76.8%) and single (29, 67.5%), and the average number of working years as a nurse and average tenure in the present ward were 6.28 and 5.47 years, respectively.

**Table 1:** The baseline characteristics of the two groups

**Item**	**EP group**	**CACP group**	**Total**	***X***^***2***^**/t**	***p***
	**n (%)**	**n (%)**	**n (%)**		
Levels of training hospitals				0.87	0.35 ^a^
Medical center	9 (47.4)	8 (33.4)	17 (39.5)		
Regional teaching hospital	10 (52.6)	16 (66.6)	26 (60.5)		
Training setting				0.18	0.91 ^a^
Medical wards	10 (52.6)	12 (50.0)	22 (51.2)		
Surgical wards	6 (31.6)	7 (29.2)	13 (30.2)		
Intensive/special units	3 (15.8)	5 (20.8)	8 (18.6)		
Number of graduating students					
Enrolled	19 (100.0)	24 (100.0)	43 (100.0)		
Completed	19 (100.0)	23 (95.8)	42 (97.7)	-	1.00 ^a, c^
Education level of mentors				1.32	0.25 ^a^
College	13 (68.4)	20 (83.3)	33 (76.8)		
University	6 (31.6)	4 (16.7)	10 (23.2)		
Marital status of mentors				0.60	0.44 ^a^
Single	14 (73.7)	15 (62.5)	29 (67.5)		
Married	5 (26.3)	9 (37.5)	14 (32.5)		
Age (yr) of mentors (means±SD)	28.84 ± 2.57	28.88 ± 2.51	28.86± 2.51	0.04	0.97 ^b^
Length of nursing working (yr)					
In all nursing settings (means±SD)	6.39 ± 2.23	6.19 ± 2.11	6.28 ± 2.14	0.30	0.77 ^b^
In current nursing setting (means±SD)	5.42 ± 2.23	5.50 ± 2.12	5.47 ± 2.14	0.12	0.90 ^b^

EP group, the externship program; CACP Group, the corporate-academic cooperation program; SD, Standard deviation; ^a^: Chi-square; ^b^: independent 2-sample t test, 2 tailed; ^c^: correction by Fisher’s exact test.

### Group comparison in nursing competence at baseline

At baseline (T0), the mean total nursing competence scores of the students based on mentor ratings in the 2 groups were 94.68 (SD=27.14) and 121.92 (SD=39.80), respectively. All 6 subscale scores in the EP group were lower than 50.0% of the possible score, ranging from 31.1% (T/C) to 47.2% (PD). The CACP group, in contrast, ranged from 43.7% (T/C) to 64.2% (PD). Some mentors evaluated the nursing competence of the training students as "cannot do". The 3 variables with the lowest percentages of total scores across the 6 subscales for the EP group were T/C, P/E and CC, and the scores on these 3 subscales relative to the possible score ranged from 31.1 to 37.0%. The rankings on the 6 subscales were comparable for the EP and CACP groups. However, students in the CACP group exhibited higher scores than students in the EP group for the total nursing competence score and in the 6 subscales. Univariate tests on the 6 subscales showed statistically significant differences between the mean scores of the EP and CACP groups, with the exception of the P/E and IPR/C subscales (Table 2).

**Table 2:** The nursing competency of graduating students in two groups at baseline and after practicum training

**Items**	**Possible score range**	**T0**			**T1**			**Difference : CACP group versus EP group Mean difference ± SE (95%CI)**		***P ***^**a**^
		**EP group (n=19) Mean ± SD**	**CACP group (n=24) Mean ± SD**	***P***	**EP group (n=19) Mean ± SD**	**CACP group (n=23) Mean ± SD**	***P***			
The total score	46-230	94.68 ± 27.14	121.92 ± 39.80	0.02*	117.63 ± 19.01	158.52 ± 23.19	0.000†	36.43 ± 7.73	(20.71 - 52.14)	0.000†
CC subscale	7-35	12.95 ± 4.50^4^	16.63 ± 6.51^4^	0.04*	16.74 ± 2.75^iii^	23.09 ± 3.88^iii^	0.005†	5.89 ± 1.22	( 3.42 - 8.37)	0.000†
T/C subscale	7-35	10.90 ± 5.87^6^	15.29 ± 7.21^6^	0.04*	15.90 ± 3.97^i^	20.48 ± 5.43^iv^	0.000†	4.93 ± 1.63	( 1.61 - 8.26)	0.005†
P/E subscale	6-30	10.47 ± 3.73^5^	13.33 ± 6.30^5^	0.09	14.00 ± 3.09^ii^	19.83 ± 4.11^i^	0.000†	4.89 ± 1.26	( 2.33 - 7.46)	0.000†
IPR/C subscale	11-55	25.16 ± 7.49^3^	29.79 ± 10.73^3^	0.12	30.84 ± 4.94^iv^	40.83 ± 5.32^ii^	0.000†	9.33 ± 1.94	( 5.39 - 13.26)	0.000†
PD subscale	10-50	23.63 ± 6.29^1^	32.08 ± 9.61^1^	0.00*	27.21 ± 4.80^v^	37.26 ± 5.83^v^	0.002†	7.79 ± 1.84	( 4.04 - 11.53)	0.000†
Leadership subscale	5-25	11.58 ± 3.12^2^	14.79 ± 4.35^2^	0.01*	12.95 ± 2.70^vi^	17.04 ± 3.23^vi^	0.000†	3.60 ± 1.10	( 1.37 - 5.84)	0.002†

CC, Clinical care; T/C, Teaching /collaboration; P/E, Planning /evaluation; IPR/C, Interpersonal relations/communication; PD, Professional development; T0, 48 hours within practicum training; T1, after practicum training; EP group, the externship program; CACP group, the corporate-academic cooperation program; ^a^: Multivariate analysis of covariance (MANCOVA) with the level of six nursing competence variable at baseline applied as a covariate; SD, Standard deviation; SE, Standard error; *: *p* < 0.05; †: *p <* 0.01; ^1, 2, 3, 4, 5, 6^: the ranking of percentage of maximum possible scores across six subscales; ^i, ii, iii, iv, v, vi^: the ranking of percentage of improvement across six subscales.

### Group comparison in nursing competence following practicum training

At T1, all students in both groups showed improvement in their total nursing competence scores, with mean scores of 117.63 (SD=19.01) and 158.52 (SD=23.19), respectively. Most mentors agreed that students had achieved the "can do much" level for the nursing competence questionnaire. Table 2 shows that the CACP group achieved higher scores than the EP group following practicum training (T1) across all 6 subscales. For further comparisons, we used the baseline levels of the 6 nursing competence subscales as covariates for control the baseline variation of 2 groups. The MANCOVA test revealed a significant difference in the mean scores of the EP and CACP groups across the 6 subscales at T1 (Wilks' lambda: *F* = 4.000, d.f. = 6, *p* = 0.005), and a comparison of the mean scores for every subscale at T1 revealed significant differences between the 2 groups: CC (*p* = 0.005), T/C (*p* = 0.000), P/E (*p* = 0.000), IPR/C (*p* = 0.000), PD (*p* = 0.002) and leadership (*p* = 0.000). Furthermore, based on pairwise MANCOVA comparisons with the baseline levels of the 6 nursing competence subscales applied as covariates, the mean difference of CACP and EP groups at T1 (e.g., differences in total nursing competence scores, including all 6 subscale scores in these 2 groups at T1) also showed significant differences (Table 2, the last column), and the power of the study to detect the multivariate effect of 2 trial groups was 0.933. Besides, we also noted that group discrepancy was apparent in the “top 3” ranking in terms of percentage of change (i.e., the mean difference of T1-T0 divided by the total subscale score) across the 6 subscales. In the EP group, the T/C, P/E and CC subscales showed the highest improvement; however, the “top 3” rankings in the CACP group were the P/E, IPR/C and CC subscales.

### Group comparison of retention rate

The retention rate declined over time in both groups. After practicum training, 19 students (100%) remained in the EP group and 23 (95.8%) remained in the CACP group, and most of the students were engaged in a clinical job. However, as time passed, more students chose to leave their first hospital of employment. In the EP group, 10 students remained in the hospital for 3 months after beginning employment, and only 8 students remained employed 1 year after beginning employment. Three months and 1 year after beginning employment, the retention rates were 52.6% and 42.1% for the EP group and 91.7% and 79.2% for the CACP group, respectively. Over time, the retention rate in the CACP group was significantly higher than that in the EP group (Table 3).

**Table 3:** The retention of graduating students at three months and one year after beginning employment

	**EP group (n=19)**		**CACP group (n=24)**			
**Item**	**n**	**(%)**	**n**	**(%)**	***X***^***2***^	***p***
Number of graduating students						
Three months after beginning employment	10	(52.6)	22	(91.7)	^-^	0.005 ^c^ †
One year after beginning employment	8	(42.1)	19	(79.2)	6.234	0.025*

EP group, the externship program; CACP group, the corporate-academic cooperation program; *: *p <* .05; †: *p* < .01; ^c^: correction by Fisher’s exact test.

## Discussion

The findings of this study indicate that students in both the CACP and externship groups showed limited nursing competence before initiating their practicum training. However, students in both groups demonstrated increased total nursing competence based on scores in all 6 subscales after practicum training, although the students in the CACP group showed greater improvement in nursing competence. The 1-year retention rate was significantly higher for the CACP group than for the EP group.

At baseline, the nursing competence scores of students in both groups, as evaluated by the mentors, were less than or similar to half of the total score, which indicates the following 3 possibilities: junior college nursing students may have limited nursing competence that needs to be strengthened; the evaluation time point (48 hours within practicum training) may have been too early for mentors to evaluate the nursing competence of students, even though mentors were provided with a list of clinical skills that students had learned in previous classroom teaching and practical training; and students in the first 48 hours of their practicum training were still in the process of assimilating themselves into the clinical area and would therefore not be able to demonstrate their competence at that time. Future studies may measure the participants' competencies at the mid-point and end of the practicum to strengthen the research design. However, the nursing competence scores of students in the CACP group were significantly higher than those in the EP group. This discrepancy indicates that CACP courses, seminars and practicum training that are jointly designed and taught by nursing faculty and nursing managers may increase nursing competence, especially in CC, T/C, PD and leadership. Thus, junior college nursing students who received instruction only from the nursing faculty appeared to be less well-prepared to develop advanced nursing competencies, such as patient care, team collaboration, participation in professional activities and leadership skills. Liu et al. reported that 29.2% of nursing faculty had less than 3 years of clinical work before being hired as nursing faculty [24]. Kuo and Kao stated that clinical nursing experience affected the teaching effectiveness of nursing faculty [25]. Accumulation of years of clinical work by a nursing faculty member is positively correlated with the learning efficacy of students. Therefore, a classroom-practicum CACP that incorporates qualified instructors from the clinical setting in curriculum teaching could enhance the connection between theoretical knowledge and clinical practice and ultimately improve the quality of teaching. This integration would lead to increased student nursing competencies. As nurse-residency programs in American emphasized, the academic-practice partnerships working together to create curriculum and learning experiences will result in a graduate better able to make the transition to the workplace [26].

Insufficient clinical experience and competence in nursing faculty is a serious issue facing nursing education. Smith emphasized that additional credentials and preparation of faculty by joining a practice are essential areas to be addressed in nursing education and suggested that a “faculty practice plan” could help faculty connect to the world of patient care and maintain or enhance clinical expertise by extending knowledge in expert nursing and care to patients [27]. The faculty practice plan, also promoted by the Taiwan Nursing Accreditation Council (TNAC), should be adopted by nursing schools in the recruiting of faculty with academic degrees to assist the faculty in bridging the teaching-practice gap and improving teaching quality. The multivariate analysis at T1 confirmed a significant improvement for students in both groups in terms of nursing competence. As described in the literature, one-on-one clinical training provides opportunities for students to acquire nursing competence and develop practice confidence [28,29]. Increased clinical exposure to acute care areas and performance of hands-on care tasks in real clinical situations allow students to integrate professional knowledge and clinical experience. This exposure develops a holistic perspective toward patient care, consolidates learning, eases the transition to new roles and encourages young nurses to stay in the nursing profession. The EP and CACP groups both had mentors who played key roles in enhancing the students’ competence during practicum training. Therefore, mentor selection, training and evaluation are critical issues that require examination. The selection criteria for mentor nurses in this study guaranteed that the recruited mentors possessed two key qualities. One quality encompassed personal characteristics such as friendliness, kind-heartedness and an eagerness to transmit knowledge and experience to the next generation. Mentors who were welcoming, supportive and willing to undertake their mentorship role also cared for, respected and valued the students as learners and team members. These attitudes enhanced the students’ sense of belonging, strengthened team cohesion and encouraged the students to remain in the practicum unit. The other mentor quality included professional characteristics such as professional knowledge, organizational skills and accountability. In patient care situations, mentors guided students through demonstration, provision of instruction and correction of the delivery of care. Specifically, mentors facilitated problem identification by demonstrating practical critical thinking skills and provided immediate assistance as needed to contribute to the students’ confidence and work experience.

Although all recruited mentors had rich clinical experience, with 5.47 years of tenure in their current wards, on average, most mentors worried about whether the graduates had quit their training because they were not qualified instructors or because they conveyed an image of an inappropriate role model (personal communication in weekly meeting). In Taiwan, approximately 4 credits or less are required in the education curriculum for a full nursing education [25]. Mentors require tools and strategies to teach students and address individual variations in learning styles. Thus, it is necessary to strengthen mentors’ instruction abilities. “Mentor training programs” may help mentors to understand effective clinical teaching strategies and skills, including the evaluation of students’ performance and the appreciation of individual and generational differences in learning styles. To enhance the effectiveness of mentorship, the provision of comprehensive in-service education training for clinical teaching skills and professional development for mentors is essential to allow mentors to be well-equipped as instructors. Furthermore, mentors should be offered resources in a timely fashion when questions, problems or dilemmas arise. Provision of consulting, support and affirmation from educators and managers can reduce the mentors’ stress. Finally, to ensure the effectiveness of mentorship, mentors must be evaluated as well. Mentor evaluations allow educational institutions to monitor the quality of mentors and help mentors to continuously improve their instruction abilities. We propose the development of multidimensional assessments, such as self-assessment by mentors, assessment by students and observations and evaluations by the clinical nursing manager and faculty members at educational institutions, for evaluating mentor performances and ensuring mentor effectiveness.

At T1, students in the CACP group achieved higher scores than those in the EP group on all nursing competence subscales, especially the P/E, IPR/C and CC subscales. As illustrated in Table 2, the scores of 6.50 (19.83-13.33) for P/E, 11.04 (40.83-29.79) for IPR/C and 6.46 (23.09-16.63) for CC in the CACP group were significantly higher than the respective scores of 3.53 (14.00-10.47), 5.68 (30.84-25.16) and 3.79 (16.74-12.95) in the EP group. Moreover, the overall retention rates of students in the CACP group at T2 and T3 were 91.7% and 79.2%, which were higher than the rate of 42.3% reported in a 2005 investigation of the National Union of Nurses Associations of the Republic of China [8] (Table 3). This success may have resulted from the following possibilities. First, we invited qualified clinical instructors to participate in the design and instruction of 3 newly added courses. This model and content of classroom-practicum cooperation may therefore be more in line with actual needs. Personal communication in weekly meetings indicated that students held positive views of the CACP, and they expressed that the CACP provided explicit demonstrations and implicit learning experiences through which they could build strong relationships with others, collect more useful information for clinical judgment, identify problems with on-site critical thinking, deliver proper care and evaluate the outcome of management. The resulting increase in self-confidence and sense of accomplishment may perpetuate a positive feedback loop and lead to further progress in P/E, IPR/C and CC nursing competencies. Additionally, CACP also improved English proficiency, including listening, speaking and reading and writing of medical terminology, charts and nursing shift reports. This finding echoes a previous study demonstrating that interpersonal relationships, tasks in nursing care and the use of professional terminology in English were the major sources of job stress in newly graduated nurses in Taiwan and were the key factors influencing graduates’ intentions to quit [12]. Hence, our findings also suggest that the low retention rates caused by stress resulting from insufficient nursing competence could be ameliorated by CACP. The P/E, IPR/C and CC nursing competencies must first be strengthened through the CACP to retain senior college students in the nursing profession. Furthermore, maintaining joint appointments at both the clinic and academic units allows faculty members and clinical staff to cooperate more closely and bridge the gap between the classroom and the workplace, which will ultimately benefit students.

Limitations that may compromise the generalizability of the findings include the small sample size with non-simultaneous, sequential interventions, the use of non-random assignment, the inclusion of all participants who were willing to practice as nurses and the exclusion of individuals who would not work or would shift to other careers after graduation. The lack of inter-rater reliability and of mentor evaluation were weak points of this study, although the researchers reached a consensus with these mentors and the nursing administrators regarding the practicum training. However, we could not control the consistency and responses of every mentor as they instructed the students.

## Conclusion

This study evaluated a classroom-practicum corporate-academic cooperation program that included the establishment of collaborative partnerships, jointly designed and taught courses and long-term mentorship. These strategies may assist senior college students in achieving superior nursing competence and maintaining a higher retention rate. The alternative, an externship program, only enhanced students’ nursing competence, and less effort was devoted to retention. The nursing industry and academia need to participate in bridging the gap between the classroom and the workplace using cooperation between classroom teaching and practicum training, to help graduating students successfully transition to independent staff nurses.

## Abbreviations

(CACP): Corporate-academic cooperation program; (CC): Clinical care; (RNs): Registered nurses; (EP): Externship program; (P/E): Planning/evaluation; (TPR/C): Interpersonal relations/communication; (PD): Professional development; (T/C): Teaching/collaboration.

## Competing interests

The authors declare that they have no competing interests.

## Authors’ contributions

This work was completed by four authors: C-NT, C-JH, K-HC, and M-FL. CN and CJ were responsible for the study conception and design. CN, CJ, KH and MF performed the data collection and data analysis. CN, CJ and KH supervised the study. CN, CJ and MF were responsible for the drafting of the manuscript. CN and MF made critical revisions to the paper. All authors read and approved the final manuscript.

## Pre-publication history

The pre-publication history for this paper can be accessed here:

http://www.biomedcentral.com/1472-6920/13/108/prepub
